# Anthrax phylogenetic structure in Northern Italy

**DOI:** 10.1186/1756-0500-4-273

**Published:** 2011-07-29

**Authors:** Giuliano Garofolo, Luigina Serrecchia, Michela Corrò, Antonio Fasanella

**Affiliations:** 1 Istituto Zooprofilattico Sperimentale della Puglia e della Basilicata, Anthrax Reference Institute of Italy-Foggia, Italy; 2 Istituto Zooprofilattico Sperimentale delle Venezie, Padua Diagnostic Laboratory- Padova, Italy

## Abstract

**Background:**

Anthrax has almost disappeared from mainland Europe, except for the Mediterranean region where cases are still reported. In Central and South Italy, anthrax is enzootic, but in the North there are currently no high risk areas, with only sporadic cases having been registered in the last few decades. Regional genetic and molecular characterizations of anthrax in these regions are still lacking. To investigate the potential molecular diversity of *Bacillus anthracis *in Northern Italy, canonical Single nucleotide polymorphism (canSNP) and Multilocus variable number tandem repeat analysis (MLVA) genotyping was performed against all isolates from animal outbreaks registered in the last twenty years in the region.

**Findings:**

Six *B. anthracis *strains were analyzed. The canSNP analysis indicates the presence of three sublineages/subgroups each of which belong to one of the 12 worldwide CanSNP genotypes: B.Br.CNEVA (3 isolates), A.Br.005/006 (1 isolates) and A.008/009 (2 isolate). The latter is the dominant canSNP genotype in Italy. The 15-loci MLVA analysis revealed five different genotypes among the isolates.

**Conclusions:**

The major B branch and the A.Br.005/006 were recovered in the Northeast region. The genetic structure of anthrax discovered in this area differs from the rest of the country, suggesting the presence of a separate and independent *B. anthracis *molecular evolution niche. Although the isolates analyzed in this study are limited in quantity and representation, these results indicate that *B. anthracis *genetic diversity changes around the Alps.

## Introduction

*Bacillus anthracis*, the etiological agent responsible for anthrax, is a Gram-positive spore-forming bacterium. The disease affects many wild and domesticated animals, and all mammals are potentially susceptible to infection [1]. Its virulence properties characterize the course of the disease, which typically is rapidly lethal for the host, particularly in herbivorous mammals [2]. Humans are less susceptible and are considered incidental hosts, but the zoonotic potential of anthrax is well known [3]. Epidemiological data demonstrate that anthrax is now rare in Italy. However, during the period 1969-1997, 27 human deaths were registered across the country [4], indicating a natural endemic area. Improved sanitation, systematic use of the vaccine by veterinarians [5] and new breeding systems have contributed to a drastic reduction in the incidence of the disease in Italy. No human deaths have been reported since 1997. Today in Italy anthrax predominantly affects livestock in pastures in the Southern regions, but is infrequent in the Northern regions [6,7].

### The study

Molecular diversity in Italian strains of *B. anthracis *was initially studied in 2005 using eight variable number tandem repeat (VNTR) loci [6], primarily to investigate strains from the Anthrax Reference Institute of Italy repository, which covers South and Central Italy. Highly resolving molecular techniques have now been developed that allow the phylo-geographic patterns of anthrax to be understood better [8,9]. Local knowledge of anthrax is vastly improved [10-12]. In recent years, outbreaks have been recorded in the Northeastern [13] and Northern [14] regions of Italy. Unfortunately, because of the ephemeral cycles of this disease, it is not always possible to establish the full extent of anthrax distribution in specific regions. However, analysis of natural anthrax epidemics can provide insights into the relationship between patterns of spread and genetic changes that have accumulated over time in these regions.

In 2005, on a typical alpine farm in the province of Bolzano, an outbreak involved two cattle, four goats and one sheep, and one suspected human case. In 2006, there was a bovine anthrax case in the province of Belluno in the Italian Alps, and in 2008 nine cattle died from anthrax in a 10 day period in the Mugello region in the province of Florence (Tuscany). One strain was isolated from each of the above outbreaks.

**Figure 1:**
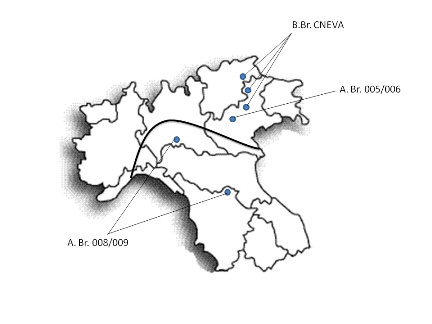
**Map of Northern Italy**. Geographical representation of the outbreaks investigated herein. The black line separates the lineage A. Br. 008/009 from the others recovered.

The phylogenetic relationship between these isolates and other archival Italian isolates was determined using 13 canonical SNPs (canSNPs) and 15 VNTR markers (MLVA15) as described by Van Ert et al. [15]. The archived samples included two strains from the province of Verona (Veneto), from outbreaks occurred in 1992 and 1995, and one from the province of Pavia (Lombardy), from a 1989 outbreak (Figure 1). These additional samples allowed a more comprehensive comparison to be carried out and provided an improved phylo-geographic pattern concerning the incidence of anthrax in northern Italy.

The analysis (Table 1) demonstrated that Northern Italy has three different sub-lineages/sub-groups of the 12 worldwide canSNP genotypes previously described by Van Ert et al. The CanSNP analysis showed that isolate 050 (Verona) belonged to sub-group 005/006 in the A lineage and that the three isolates, 049 (Verona), 187 (Bolzano) and 188 (Belluno), belonged to the sub-group B.Br. CNEVA in the smaller B lineage. Isolates, 187 and 188, were isolated at different sites but have the same MLVA type, although they differ from isolate 049 from Verona (Table 2). The isolates 145 (Florence) and 183 (Pavia) belonged to CanSNP sub-group A.Br.008/009 but had two different MLVA types (Tables 1 and 2).

**Table 1:** *B. anthracis *isolates used in this study and their genotypes based on 13 Canonical SNPs

Isolate name	Locality (Province)	canSNP lineage/group	**A**. **Br**. 001	**A**. Br. 002	**A**. Br. 003	**A**. Br. 004	**A**. Br. 006	**A**. Br. 007	**A**. Br. 008	**A**. Br. 009	**B**. Br. 001	**B**. Br. 002	**B**. Br. 003	**B**. Br. 004	**A/B**. **Br**. 001
049	Verona	B.Br.CNEVA	T	G	A	T	C	T	T	A	T	G	A	C	A
187	Bolzano	B.Br.CNEVA	T	G	A	T	C	T	T	A	T	G	A	C	A
188	Belluno	B.Br.CNEVA	T	G	A	T	C	T	T	A	T	G	A	C	A
050	Verona	A.Br.005/006	T	G	A	T	A	T	T	A	T	G	G	T	A
145	Florence	A.Br.008/009	T	G	A	T	A	T	G	A	T	G	G	T	A
183	Pavia	A.Br.008/009	T	G	A	T	A	T	G	A	T	G	G	T	A

**Table 2:** MLVA genotypes of *B. anthracis *isolates used in this study

								**VNTR**^**$**^								
**Isolate name**	**MLVA type**	**vrrb1**	**vrra**	**vrrb2**	**pX01**	**vrrc1**	**pX02**	**vrrc2**	**cg3**	**vntr16**	**vntr19**	**vntr32**	**vntr12**	**vntr17**	**vntr23**	**vntr35**

049	I	9	8	7	5	7	5	3	3	20	4	13	6	2	5	5
187	II	5	8	7	6	7	5	3	3	18	4	13	6	2	5	5
188	II	5	8	7	6	7	5	3	3	18	4	13	6	2	5	5
050	III	10	10	8	5	8	6	5	3	17	4	10	7	3	5	4
145	IV	10	9	7	6	8	6	5	2	16	4	12	7	3	6	4
183	V	10	9	7	5	8	6	5	2	16	4	12	7	4	6	4

^$^Numbers of tandem repeats in each locus for *Bacillus anthracis *isolates are reported. The copy numbers for each locus are calculated as (amplicon size -offset)/repeat size. The amplicon sizes were determined and calculated using ABI 3130 genetic analyzer and rounded with GeneMapper software using a binning code from the Italian genotype database. Offset and repeat sizes are available online [18]http://minisatellites.u-psud.fr/GPMS/.

## Discussion and conclusion

Fasanella et al. [6] suggested that the dominant phylogenetic group in Italy belongs to the previously described Cluster A1.a [16], which is now classified as part of the sub-group A.Br.008/009 [15], also known as the Trans Euro Asian (TEA) group, in the A lineage. Our datum is consistent with the overall presence of this particular sub-group throughout the majority of Europe and regions of Asia [10]. In the regions of Tuscany and Lombardy, the TEA group was recovered indicating that it belongs to the major Italian sub-group. We also identified two new MLVA genotypes (Table 2) which are not present in the Italian database (data not shown). While these relatively "modern" isolates do not necessarily represent a diverse A.Br.008/009 sub-group, they do reinforce the hypothesis that the TEA sub-group in Italy constitutes part of the historical spread of anthrax across Europe and Asia [10].

The results also demonstrate that in a relatively small area in Northeast Italy, unique genotypes were recovered from the major *B. anthracis *worldwide clonal A and B lineages. These isolates were distinctive from the A.Br.008/009 TEA branch that dominates throughout Europe and Asia. These data suggest that the Italian Alps may share a unique environmental niche and phylo-geographic pattern representative of the B.Br. CNEVA sub-group (previously identified as B2 cluster [16]). This proposition is supported by reports that this lineage is present in the French Alps [17], Germany and Croatia [15]. Isolates from the B.Br.CNEVA (B2) sub-group are almost entirely restricted to Europe. This may indicate the presence of a common environment or corridor that could have allowed this unusual lineage to persist in livestock and wildlife in the French and Italian Alps. A small number of naturally occurring isolates may represent previous "anthrax regions", where a lineage may have continued to recycle over long periods of time despite the lack of incidents in the more visible domesticated populations. In addition, the identification of two new MLVA15 types among the three strains analyzed from the B.Br. CNEVA lineage with three alleles of difference may suggest a longer history for this lineage in the area, although only SNP analysis based on whole genome sequencing could confirm this hypothesis.

More puzzling is the recent discovery of the A.Br. 005/006 sub-group in Northeast of Italy, which suggests that the history of anthrax in Italy is complex and is likely to have involved the introduction and re-introduction of this disease through repeated human activity led by the trading of commodities. This sub-group is well represented in Africa but rare in Europe. Its presence in the Northeastern territories of Italy could be related to trade exchanges dating back to the Maritime Republics period (Middle Ages), when city states competed for trade and commerce throughout the Mediterranean. This highlights the possibility that this rare, predominantly African, lineage could have become present in this small region of Italy owing to an oddity in how goods were traded from Africa to Northern Italy.

It is particularly interesting that, in a small area of Northeast Italy, *B. anthracis *presents with major genetic variability, suggesting the presence of a different environment that permits rare and unusual lineages to survive.

## Competing interests

The authors declare that they have no competing interests.

## Authors' contributions

GG conceived the study, coordinated the molecular genetic studies and drafted the manuscript. LS performed the 15-loci MLVA and CanSNP analyses. MC coordinated the collection of samples. AF designed the study and revised the manuscript. All authors read and approved the final manuscript.
